# K-homology splicing regulatory protein (KSRP) promotes post-transcriptional destabilization of Spry4 transcripts in non-small cell lung cancer

**DOI:** 10.1074/jbc.M116.757906

**Published:** 2017-03-08

**Authors:** Rama Kamesh Bikkavilli, Sereke Adam Zerayesus, Michelle Van Scoyk, Lora Wilson, Pei-Ying Wu, Abhinaya Baskaran, Ke Tang, Syed Raheem, Blain A. Samuelson, Narsa M. Reddy, Sekhar P. Reddy, Carlyne D. Cool, Beata Kosmider, Sreedevi Avasarala, Robert A. Winn

**Affiliations:** From the ‡ Division of Pulmonary, Critical Care, Sleep and Allergy, Department of Medicine and; ¶ Division of Developmental Biology and Basic Research, Department of Pediatrics,University of Illinois, Chicago, Illinois 60612,; § Department of Pathology and Division of Pulmonary Sciences and Critical Care Medicine, School of Medicine, University of Colorado Anschutz Medical Campus, Aurora, Colorado 80045,; ‖ Departments of Physiology, Thoracic Medicine, and Surgery, Lewis Katz School of Medicine and; ** Center for Inflammation, Translational, and Clinical Lung Research, Temple University, Philadelphia, Pennsylvania 19140,; ‡‡ Department of Medicine, National Jewish Health, Denver, Colorado 80206, and; §§ Jesse Brown Veterans Affairs Medical Center, Chicago, Illinois 60612

**Keywords:** cell invasion, cell migration, mRNA decay, post-transcriptional regulation, RNA-binding protein, KSRP, NSCLC, Spry4

## Abstract

AU-rich element-binding proteins (ARE-BPs) offer post-transcriptional regulation of gene expression via physical interaction and recruitment of RNA decay machinery to the AU-rich elements within the 3′-UTR of the target transcripts. However, the role of ARE-BPs in lung cancer remains poorly understood. In this study, we have identified that K-homology splicing regulatory protein (KSRP), an ARE-BP, is robustly up-regulated in human lung cancer. Importantly, Kaplan-Meier survival analysis indicated that elevated KSRP expression was correlated with poor overall survival of lung cancer patients. Furthermore, cigarette smoke, a leading risk factor for lung cancer, was also identified to be an important contributor to increased KSRP expression. Remarkably, silencing of KSRP decreased cell proliferation, reversed anchorage-independent growth, and reduced migration/invasion, suggesting an oncogenic role for KSRP in lung cancer. Finally, we provide mechanistic evidence that KSRP promotes the down-regulation of Spry4 by a previously unidentified mechanism, *i.e.* post-transcriptional mRNA regulation.

## Introduction

Lung cancer, which primarily arises from lung epithelial cells, is the leading cause of cancer deaths for both men and women in the United States (1, 2). Importantly, the numbers of lung cancer deaths are more than breast, prostate, and colorectal cancers combined (1, 2). Despite advances in early detection and the development of molecular targeted therapies, which have shown some promise (3), a large number of mortalities still persists. Hence, identification of additional molecular targets is a research priority in lung cancer therapeutic research.

AU-rich element-binding proteins (ARE-BPs)^2^ offer post-transcriptional regulation of gene expression via physical interaction and recruitment of RNA decay machinery to the AU-rich elements within the 3′-untranslated region (UTR) of the target transcripts (4–6). The mRNAs with AU-rich elements (AREs) in their 3′-UTR constitute ∼10–15% of all the transcripts that span genes related to inflammation, transcription, cell proliferation, RNA metabolism, development, and cellular signaling, which play important roles in tumorigenesis (4–6). However, the role of ARE-BPs in lung cancer remains poorly understood. K-homology splicing regulatory protein (KSRP) belongs to the family of ARE-BPs, which promote rapid decay of select ARE-containing mRNAs (5, 6). Of note, KSRP has never been previously associated with lung cancer.

In this study, we observed a strong up-regulation in the expression of KSRP by using The Cancer Genome Atlas (TCGA) lung cancer data sets. To fully appreciate the functional role of KSRP in lung tumorigenesis, first we validated the expression of KSRP in lung cancer tissue microarrays (TMAs) and a panel of non-small-cell lung cancer (NSCLC) cell lines. We also identified cigarette smoke, a leading risk factor for lung cancer, as an important contributor for increased KSRP expression. Finally, by utilizing a battery of cell-based assays, we demonstrate a novel function for KSRP in the regulation of transformed cell growth through the down-regulation of Spry4, a tumor suppressor protein, by a previously unidentified mechanism, *i.e.* post-transcriptional regulation.

## Results

### Expression of KSRP in human lung cancer and NSCLC cell lines

**Figure 1.**
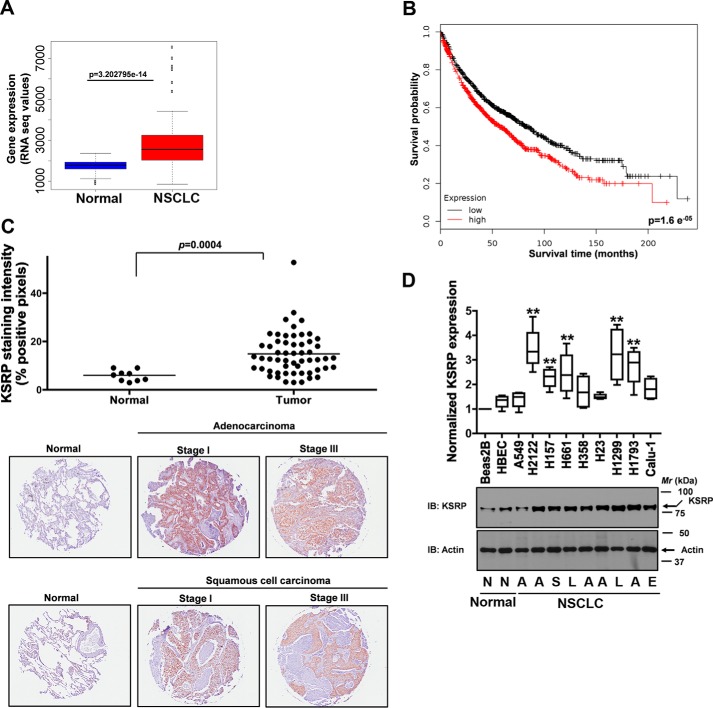
**KSRP is up-regulated in lung cancer.** *A*, analysis of publicly available TCGA lung cancer data sets revealed a significant up-regulation in KSRP expression in lung cancer. *B*, online survival analysis software (Kaplan-Meier plotter) was used to assess the prognostic value of KSRP expression in lung cancer. *C*, KSRP staining of a TMA that includes normal and lung cancer tissue sections. The u*pper panel* represents the staining intensity of KSRP, whereas representative images are displayed in the *lower panel. p* = 0.0004, *t* test. *D*, cell lysates of human non-transformed bronchial epithelial cells (Beas2B and HBEC) and a panel of NSCLC cell lines were probed for the expression of KSRP using anti-KSRP antibodies. *N*, normal; *A*, adenocarcinoma; *S*, squamous carcinoma; *L*, large cell carcinoma; *E*, epidermoid carcinoma. *Error bars* represent mean ± S.E. **, *p* < 0.01, ANOVA. *RNA seq*, RNA sequencing.

To determine the expression of KSRP in human lung tumors, we downloaded the lung cancer data sets from TCGA. The lung cancer data sets comprise 103 normal-tumor matched samples. The expression of KSRP is considered to be up-regulated if the Wald statistics value of the differential expression (normal *versus* tumor) is greater than 0 with a significant *p* value (<0.05), and the expression is considered to be down-regulated if the Wald statistics value is less than 0 with a significant *p* value (<0.05). A strong up-regulation of KSRP expression was observed in lung cancer data sets (Fig. 1*A*). Additionally, we performed Kaplan-Meier survival analysis by using the Kaplan-Meier plotter, an online tool (7). Our analyses indicated that higher KSRP expression was correlated with poor overall survival of lung cancer patients (Fig. 1*B*). To corroborate our *in silico* findings, we next looked for the expression of KSRP in human lung cancer specimens via staining lung cancer TMA slides (Biomax) with KSRP-specific antibodies followed by image analysis using a positive pixel count algorithm (Aperio). The TMA included 90 cases of lung carcinoma and 10 normal tissues in duplicate cores per case. Of note, the normal tissues were not matched samples. Consistent with our *in silico* findings, KSRP expression was significantly up-regulated in lung cancer tissues when compared with normal tissues (Fig. 1*C*). Representative immunohistochemistry images of normal lung, lung adenocarcinoma, and lung squamous carcinoma are shown in Fig. 1*C*. Finally, the expression of KSRP was also evaluated in non-transformed bronchial epithelial cell lines (Beas2B and human bronchial epithelial cell line (HBEC)) and NSCLC cell lines via immunoblotting (Fig. 1*D*). In agreement with our *in silico* analysis and TMA data, up-regulation in KSRP expression was also observed in 56% of NSCLC cell lines evaluated (Fig. 1*D*). Collectively, we show for the first time that KSRP expression is significantly up-regulated in lung cancer.

### Effects of cigarette smoke on KSRP expression

Because cigarette smoking is a leading cause for lung cancer, we evaluated the effects of cigarette smoke (CS) on KSRP expression. For these studies, we first measured KSRP protein expression in the lung tissue samples from people who never smoked (non-smokers) and from people who smoked 10–25 cigarettes per day for at least 3 years (smokers). When compared with non-smokers, KSRP expression was significantly elevated in smokers (supplemental Fig. 1A). We also assessed the effects of CS exposure on KSRP expression in C57/BL6 mice (supplemental Fig. 1B). Consistent with the observations made in human tissues, a significant increase in KSRP expression was also observed in the lung tissue samples of mice exposed to CS for 6 months (supplemental Fig. 1B). To further confirm our observation that CS induced the expression of KSRP, we exposed Beas2B cells to cigarette smoke condensate (CSC). Exposure of Beas2B cells to CSC also induced KSRP expression (supplemental Fig. 1C). Taken together, these data suggest that CS is an important contributing factor for increased KSRP expression.

### Effects of KSRP knockdown on cell proliferation

**Figure 2.**
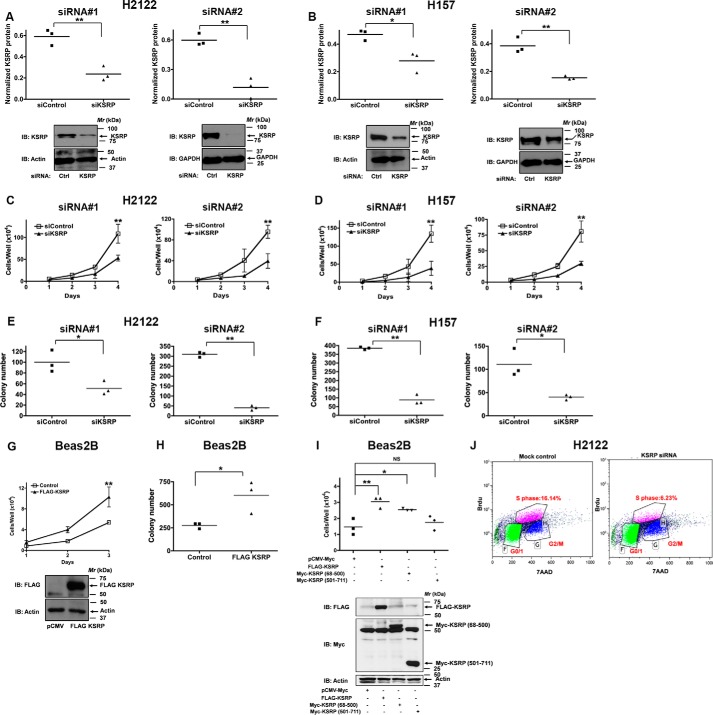
**KSRP regulates NSCLC cell proliferation.** *A* and *B*, H2122 and H157 cells were treated with either control siRNAs or KSRP-specific siRNAs, and the extent of KSRP knockdown was determined by immunoblotting. *, *p* < 0.05; **, *p* < 0.01, *t* test. *C* and *D*, H2122 and H157 cells were treated with KSRP-specific siRNAs, and the effects of KSRP knockdown on cell proliferation were determined by hemocytometer cell counting. *Error bars* represent mean ± S.E. **, *p* < 0.01, ANOVA. *E* and *F*, H2122 and H157 cells were treated with KSRP-specific siRNAs, and the effects of KSRP knockdown on cell proliferation were determined by clonogenic assays. *, *p* < 0.05; **, *p* < 0.01, *t* test. *G* and *H*, Beas2B cells were transiently transfected with either control or FLAG-KSRP (amino acids 68–711) expression vectors, and the proliferation rates of the cells were determined by hemocytometer cell counting (*error bars* represent mean ± S.E; **, *p* < 0.01, ANOVA) and clonogenic assays (*, *p* < 0.05, *t* test). *I*, Beas2B cells were transiently transfected with control, FLAG-KSRP (amino acids 68–711), Myc-KSRP(68–500), and Myc-KSRP(501–711) expression vectors, and the proliferation rates of the cells were determined by hemocytometer cell counting. *, *p* < 0.05; **, *p* < 0.01, ANOVA. *J*, mock-transfected or KSRP siRNA-transfected H2122 cells were pulse-labeled with BrdU. The cell proliferation rates were later determined by staining with FITC-anti-BrdU antibodies followed by flow cytometry as described under “Experimental procedures.” Representative data of two independent, highly reproducible experiments are displayed. *Ctrl*, control; *IB*, immunoblotting; *7AAD*, 7-aminoactinomycin D.

Having established an association of increased KSRP expression in lung cancer, we next sought to identify the role of KSRP in driving cell proliferation by using KSRP-specific small interfering RNAs (siRNAs) and short hairpin RNAs (shRNAs) in NSCLC cells (Fig. 2). Because KSRP is up-regulated in NSCLC, we chose to study the effects of KSRP knockdown on cell proliferation in several NSCLC cell lines, *e.g.* H2122 (derived from lung adenocarcinoma) and H157 (derived from lung squamous carcinoma). Transfection of H2122 and H157 with two different KSRP-specific siRNAs resulted in a significant reduction of KSRP expression when compared with cells transfected with scrambled siRNA controls (Fig. 2, *A* and *B*). Under similar knockdown conditions, a significant reduction in cell proliferation rates was observed in H2122 and H157 cells as determined by hemocytometer cell counting (Fig. 2, *C* and *D*) and clonogenic cell proliferation assays (Fig. 2, *E* and *F*). Additionally, we assessed cell proliferation via bromodeoxyuridine (BrdU) incorporation (Fig. 2*J*). The proportion of KSRP siRNA-treated H2122 cells in S phase (6%) was lower than the proportion of mock-transfected H2122 cells (16%), indicating that silencing KSRP expression suppressed the proliferation of H2122 cells (Fig. 2*J*). To further explore the role of KSRP in cell proliferation, we examined the effects of KSRP overexpression in Beas2B cells (Fig. 2, *G* and *H*). For these studies, a FLAG-tagged version of human KSRP (amino acids 68–711) was used in transient transfections of Beas2B cells (Fig. 2*G*), and the expression of FLAG-KSRP was ascertained by immunoblotting (Fig. 2*G*). Forced overexpression of KSRP in Beas2B cells resulted in an increased cell proliferation as determined by hemocytometer cell counting (Fig. 2*G*) and clonogenic cell proliferation assays (Fig. 2*H*). We next sought to identify the region of KSRP that promotes cell proliferation (Fig. 2*I*). Expression of the N terminus of KSRP harboring the K-homology motifs (Myc-KSRP(68–500)), but not the C terminus of KSRP (Myc-KSRP(501–711)), promoted Beas2B cell proliferation (Fig. 2*I*). These observations suggest that KSRP promotes cell proliferation via its N terminus, which encodes K-homology domains.

### Effects of KSRP knockdown on anchorage-independent cell growth

**Figure 3.**
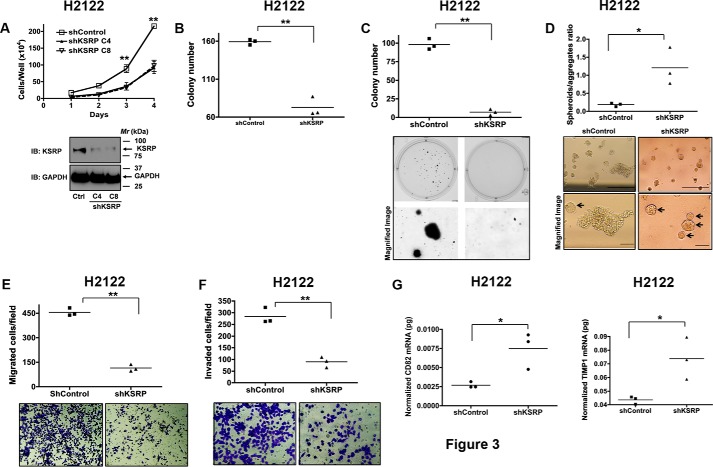
**KSRP regulates anchorage-independent growth, cell migration, and invasion.** *A* and *B*, clones with stable expression of control shRNA or KSRP shRNA were generated as described under “Experimental procedures.” The cell proliferation rates of the stable clones were later determined by hemocytometer cell counting (*A*; *error bars* represent mean ± S.E.; **, *p* < 0.01, ANOVA) or clonogenic assays (*B*; **, *p* < 0.01, *t* test). *C*, anchorage-independent growth of H2122 clones with stable expression of either control shRNA or KSRP shRNA was assayed using soft agar assays as described under “Experimental procedures.” **, *p* < 0.01, *t* test. *D*, H2122 clones stably expressing either control shRNA or KSRP shRNA were embedded in Matrigel as single cells as described under “Experimental procedures.” The number of spheroids (*arrows*) and aggregates were counted and represented in the graph. *Scale bar*, 200 μm. *, *p* < 0.05, *t* test. *E*, migration of H2122 clones with stable expression of either control shRNA or KSRP shRNA was assayed in Transwell inserts as described under “Experimental procedures.” The *top panel* represents the number of cells migrated, whereas representative images are displayed in the *bottom panel*. **, *p* < 0.01, *t* test. *F*, invasion of H2122 clones with stable expression of either control shRNA or KSRP shRNA was assayed in Transwell inserts coated with Matrigel as described under “Experimental procedures.” The *top panel* represents the number of cells that invaded, whereas representative images are displayed in the *bottom panel*. **, *p* < 0.01, *t* test. *G* and *H*, total RNA from H2122 clones with stable expression of either control shRNA or KSRP shRNA was isolated, and the expression of CD82 (*G*) and TIMP1 (*H*) was later evaluated by qPCR as described under “Experimental procedures.” *, *p* < 0.05, *t* test. *Ctrl*, control; *IB*, immunoblotting.

To determine the effects of KSRP knockdown on anchorage-independent cell growth, we developed multiple H2122 clones with stable expression of either control shRNA or KSRP shRNA to exclude the effects of clonal variation. Consistent with the effects of KSRP siRNAs on cell proliferation; stable knockdown of KSRP in H2122 cells also resulted in reduced cell proliferation as determined by hemocytometer cell counting (Fig. 3*A*) and clonogenic cell proliferation assays (Fig. 3*B*). The soft agar colony formation assay is a commonly used tool to measure anchorage-independent growth, a strict measure of transformed cell growth (8). To evaluate the effects of KSRP knockdown on anchorage-independent growth, H2122 clones stably expressing either control shRNA or KSRP shRNA were seeded into soft agar cultures. KSRP knockdown resulted in a remarkable reduction in the number of colonies formed when compared with H2122 clones stably expressing control shRNAs (Fig. 3*C*).

Three-dimensional (3D) cultures allow phenotypic differentiation of non-malignant cells from malignant cells: non-malignant cells form highly organized spheroid-like structures, whereas malignant cells form unorganized, poorly proliferative structures. To examine whether KSRP plays a role in the phenotypic differentiation, H2122 clones stably expressing KSRP shRNAs were embedded in Matrigel matrices as single cells. As illustrated in Fig. 3*D*, KSRP knockdown induced spheroid formation similar to that of non-malignant cells (Fig. 3*D*), whereas control shRNA-treated cells formed poorly differentiated structures, a characteristic of malignant cells (Fig. 3*D*).

### Effects of KSRP knockdown on migration and invasion

To evaluate the possible role of KSRP in NSCLC cell migration, we performed cell migration assays using Transwell (8-μm pore size) cell culture inserts. It was interesting to note that the stable knockdown of KSRP resulted in reduced cell migration when compared with H2122 clones stably expressing control shRNAs (Fig. 3*E*). We also evaluated the invasive abilities of H2122 clones stably expressing KSRP shRNAs by seeding the cells into Transwell cell culture inserts coated with Matrigel. The cells that invaded the Matrigel and migrated through the pores were later stained and counted and are represented in Fig. 3*F*. When compared with H2122 clones expressing control shRNAs, H2122 clones with stable knockdown of KSRP also displayed reduced invasive abilities (Fig. 3*F*). It was shown earlier that CD82-TIMP1 signaling suppresses tumor invasion via the inactivation of MMP9 (9). In accordance with the effects of KSRP knockdown on cell invasion, we also observed a significant increase in CD82 and TIMP1 expression in H2122 clones stably expressing KSRP shRNA (Fig. 3, *G* and *H*).

### KSRP is a novel regulator of Spry4 tumor suppressor protein

**Figure 4.**
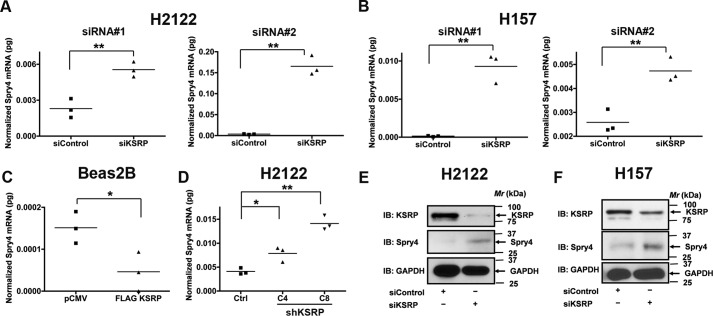
**KSRP is a novel regulator of Spry4.** *A* and *B*, total RNA from H2122 and H157 cells treated with KSRP-specific siRNAs was isolated, and the expression of Spry4 was later determined by qPCR as described under “Experimental procedures.” **, *p* < 0.01, *t* test. *C*, total RNA from Beas2B cells transiently transfected with either control or FLAG-KSRP expression vectors was isolated, and the expression of Spry4 was later determined by qPCR as described under “Experimental procedures.” *, *p* < 0.05, *t* test. *D*, total RNA from H2122 clones with stable expression of control shRNA or KSRP shRNA was isolated, and the expression of Spry4 was later determined by qPCR as described under “Experimental procedures.” *, *p* < 0.05; **, *p* < 0.01, ANOVA. *E* and *F*, proteins from H2122 and H157 cells treated with KSRP-specific siRNAs were isolated, and the expression of Spry4 was later determined by immunoblotting. Representative data of two independent, highly reproducible experiments are displayed. *Ctrl*, control; *IB*, immunoblotting.

We chose to interrogate the role of KSRP in the regulation of Spry4 for the following reasons. 1) Spry4 has been previously shown to regulate NSCLC transformed cell growth, migration, and invasion (9). 2) Microarray data on KSRP-associated mRNAs showed enrichment of Spry4 (10). 3) *In silico* analysis of the 3′-UTR of Spry4 mRNA revealed eight class I (AUUUA) AREs. Because ARE-containing mRNAs are targeted by KSRP for degradation, we postulated that KSRP might represent a novel regulator of Spry4. To resolve this question, we first examined the mRNA expression of Spry4 in H2122 and H157 cells following siRNA-mediated KSRP knockdown (Fig. 4). Treatment of H2122 and H157 cells with KSRP siRNAs resulted in a robust increase in the expression of Spry4 transcripts (Fig. 4, *A* and *B*). Identical results were obtained in H2122 clones with stable knockdown of KSRP (Fig. 4*D*). Forced overexpression of KSRP in a non-transformed bronchial epithelial cell line, *i.e.* Beas2B, resulted in a significant reduction in the expression of Spry4 mRNA levels (Fig. 4*C*). Likewise, treatment of H2122 and H157 cells with KSRP siRNAs also resulted in a significant increase in Spry4 protein (Fig. 4, *E* and *F*).

### KSRP binds to the 3′-UTR of Spry4 mRNA

**Figure 5.**
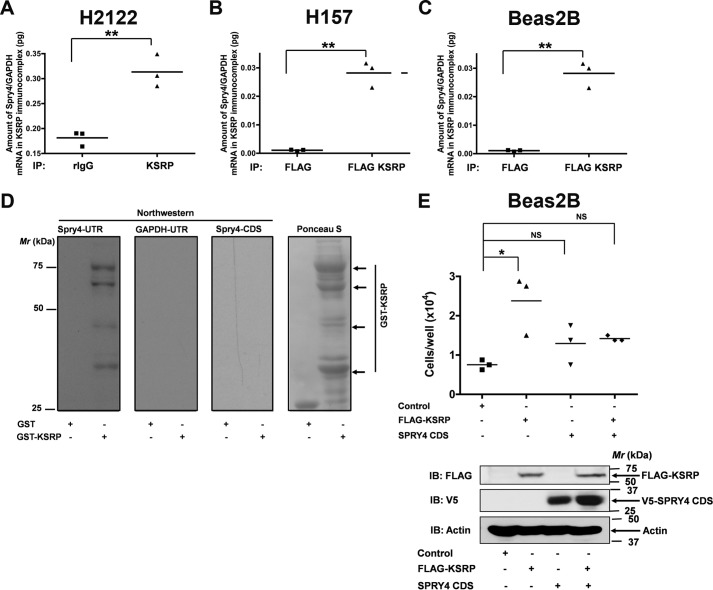
**KSRP binds to Spry4 3′-UTR.** *A* and *B*, RNA immunoprecipitation assay was performed on H2122 (*A*) and H157 (*B*) cell lysates with either control IgG or KSRP antibodies as described under “Experimental procedures.” The amounts of Spry4/GAPDH mRNA in the KSRP immunoprecipitates were later quantified using qPCR. *, *p* < 0.05; **, *p* < 0.01, *t* test. *C*, RNA immunoprecipitation assay was performed on the lysates of Beas2B cells expressing FLAG-KSRP with anti-FLAG antibodies. The amounts of Spry4/GAPDH mRNA in the KSRP immunoprecipitates were later quantified using qPCR. **, *p* < 0.01, *t* test. *D*, Northwestern analysis of KSRP and Spry4 UTR. Recombinant GST and GST-KSRP were separated by SDS-PAGE and transferred onto nitrocellulose membrane. Northwestern analysis was then performed either using DIG-labeled Spry4 UTR, GAPDH UTR, or *Spry4* CDS probes as described under “Experimental procedures.” GAPDH UTR and *Spry4* CDS probes were used as negative controls. Representative data of two independent, highly reproducible experiments are displayed. *E*, Beas2B cells were co-transfected with FLAG-KSRP (amino acids 68–711) and *Spry4* CDS expression vectors. The proliferation rates of the cells were determined after 24 h by hemocytometer cell counting. KSRP-induced proliferative effects were attenuated upon co-expression of *Spry4* CDS, suggesting that the proproliferative effects of KSRP were mediated through the down-regulation of Spry4. *, *p* < 0.05, ANOVA. *IP*, immunoprecipitation; *IB*, immunoblotting; *rIgG*, rabbit IgG; *NS*, not significant.

Because KSRP-mediated mRNA regulation involves the physical interaction of KSRP with the ARE-containing mRNAs, we evaluated whether KSRP was able to bind directly to Spry4 mRNA in a series of experiments (Fig. 5). To this end, we first performed RNA immunoprecipitations. In these experiments, KSRP was first immunoprecipitated from H2122 and H157 total cell lysates. Later, total RNA from the KSRP immunoprecipitate was isolated, and the amount of Spry4 and GAPDH transcripts in the RNA was evaluated by reverse transcription (RT)-qPCR (Fig. 5, *A* and *B*). This procedure has been successfully used in the past to demonstrate the interaction of KSRP with ARE-containing mRNAs (11). Increased enrichment of Spry4/GAPDH transcripts was detected in the KSRP immunocomplexes (Fig. 5, *A* and *B*). Similar results were obtained in an independent experiment wherein FLAG-KSRP was immunoprecipitated with anti-FLAG antibodies, indicating the validity of our assay (Fig. 5*C*).

The interaction between KSRP and Spry4 was also investigated using Northwestern analysis. For these studies, recombinant GST or GST-KSRP proteins transferred onto nitrocellulose membranes were probed with *in vitro* transcribed digoxigenin (DIG)-labeled full-length Spry4 3′-UTR (bases 1160–4941). Spry4 UTR probe demonstrated binding to GST-KSRP but not to GST alone (Fig. 5*D*), whereas the DIG-labeled GAPDH 3′-UTR probe and *Spry4* coding sequence (CDS) probe, tested as negative controls, did not bind to GST-KSRP (Fig. 5*D*). Because the GST-KSRP fusion protein was partially purified, we believe that the multiple bands were the result of endoproteolytic cleavage of GST-KSRP fusion protein in bacteria.

To determine whether KSRP promotes cell proliferation via the down-regulation of Spry4, we co-transfected human non-transformed bronchial epithelial cells (Beas2B) with KSRP and *Spry4* CDS (*Spry4* coding sequence that cannot be regulated by KSRP; Fig. 5*D*). Significantly, KSRP-induced proliferative effects were attenuated upon co-expression of *Spry4* CDS (Fig. 5*E*), suggesting that the proproliferative effects of KSRP were mediated through the down-regulation of Spry4. These data provide a mechanistic link between KSRP and Spry4 in the context of the regulation of cell proliferation.

### KSRP promotes post-transcriptional destabilization of Spry4 mRNA

**Figure 6.**
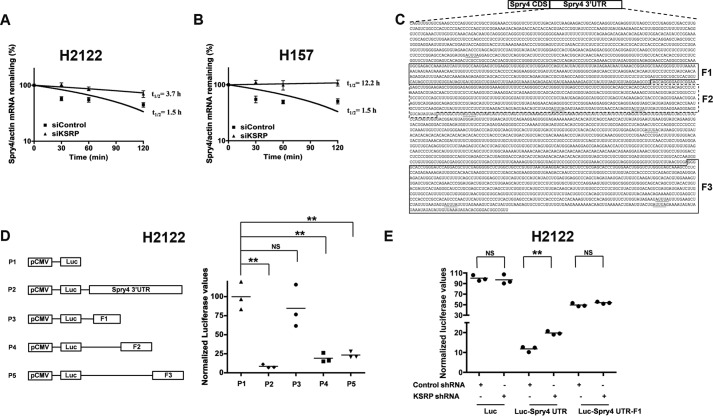
**KSRP promotes post-transcriptional destabilization of Spry4 transcripts.** *A* and *B*, H2122 (*A*) and H157 (*B*) cells were transfected with either control or KSRP siRNAs followed by treatment with actinomycin D for the indicated periods of time. Spry4 and actin mRNA levels were quantified using qPCR, and the amounts of Spry4/actin mRNA remaining were plotted on a semilogarithmic scale. *Error bars* represent mean ± S.E. **, *p* < 0.01, ANOVA. *C*, schematic representation of the Spry4 mRNA with 3′-UTR displaying class I AREs. *D*, H2122 cells were transiently transfected with pcDNA-Luc or pcDNA-Luc with either full-length Spry4 3′-UTR (bases 1160–4961) or Spry4 3′-UTR truncations F1 (bases 2012–2650), F2 (bases 2651–3006), and F3 (bases 4072–4941). Later, the luciferase activities in the cell lysates were assayed using a luminometer, and normalized luciferase values are displayed in the graph. **, *p* < 0.01, ANOVA. *E*, H2122 cells were co-transfected with the indicated plasmids. Later, the luciferase activities in the cell lysates were assayed using a luminometer, and normalized luciferase values are displayed in the graph. **, *p* < 0.01, ANOVA. *NS*, not significant.

KSRP is an mRNA decay-promoting factor, which promotes rapid decay of ARE-containing mRNAs (11). We therefore aimed to determine the influence of KSRP knockdown on Spry4 mRNA stability. For these studies, H2122 and H157 cells were transiently transfected with KSRP siRNAs followed by the treatment with actinomycin D to block *de novo* mRNA synthesis. The cells were harvested at various times (0, 30, 60, and 120 min) after actinomycin D treatment. The half-lives of Spry4 mRNA were later determined by RT-PCR. In H2122 and H157 cells, Spry4 mRNA displayed significant mRNA decay (Fig. 6, *A* and *B*). Treatment of H2122 and H157 cells with KSRP siRNAs, on the contrary, stabilized Spry4 mRNA as demonstrated by an increase in the half-life of Spry4 mRNA (Fig. 6, *A* and *B*).

To evaluate the functional significance of the 3′-UTR of Spry4 mRNA (Fig. 6*C*), we developed a chimeric luciferase-Spry4 3′-UTR construct wherein the Spry4 3′-UTR was cloned downstream of firefly luciferase gene. If the Spry4 3′-UTR was functionally significant, it is anticipated that RNA-binding proteins, *e.g.* KSRP, would repress luciferase expression by targeting Spry4 3′-UTR. Indeed, the presence of Spry4 3′-UTR (Fig. 6*D*, *P2*) significantly reduced the expression of luciferase when compared with the luciferase reporter without Spry4 3′-UTR (Fig. 6*D*, *P1*). We next sought to identify the regulatory region within the Spry4 3′-UTR. For these experiments, we focused on the regions of Spry4 3′-UTR with clustered AREs. Dissection of the Spry4 3′-UTR revealed that bases 2651–4941 of the Spry4 3′-UTR (F2 and F3 regions) are functionally important (Fig. 6*D*, *P4* and *P5*). It is noteworthy to mention that six class I (AUUUA) AREs were encoded within the F2 and F3 regions of Spry4 3′-UTR. These data indicate that Spry4 transcripts encode a functional 3′-UTR.

To ascertain whether the effects of KSRP on Spry4 3′-UTR were specific, we performed studies using a luciferase-Spry4 3′-UTR chimeric construct and KSRP shRNAs. If the effects of KSRP on Spry4 3′-UTR were specific, then upon KSRP knockdown the KSRP-mediated mRNA repression should be relaxed as detected by an increase in luciferase activities (Fig. 6*E*). Indeed, knockdown of KSRP increased the luciferase activities of Luc-Spry4 UTR but not the luciferase activities of Luc-Spry4 UTR-F1 (Fig. 6*E*). The small increase in the luciferase activities in the presence of KSRP shRNAs can be attributed to the efficacy of KSRP knockdown in these experiments.

## Discussion

This is the first report demonstrating a novel mechanism of Spry4 regulation via a post-transcriptional mechanism mediated by KSRP. Spry4 is an important regulator of receptor tyrosine kinase signaling, which plays an essential role during growth, differentiation, and tumorigenesis (9, 12). Of note, Spry was shown to be an important negative regulator of protumorigenic fibroblast growth factor (FGF) and epidermal growth factor (EGF) signaling pathways (9, 12), underscoring the importance of this molecule and the newly identified mechanism in tumor suppression. Spry proteins are frequently down-regulated in cancers (9, 12); however, the mechanism of Spry4 down-regulation remains incompletely understood. Decreased mRNA expression can be attributed to reduced transcription and/or increased mRNA degradation. In the current study, we demonstrate that the loss of Spry4 in lung cancers is due to increased Spry4 mRNA degradation as a result of up-regulated KSRP expression.

KSRP is an ARE-BP that specifically binds and recruits RNA decay machinery to ARE-containing transcripts, subjecting them to degradation (13, 14). The importance of KSRP in several developmental processes has been highlighted (6, 11, 13–24, 26–40). However, the role of KSRP in lung cancer has not been previously demonstrated. In the current study, we show that KSRP is robustly up-regulated in lung cancer (Fig. 1). Subsequently, we also highlight the importance of KSRP in cell proliferation, migration/invasion, and transformed cell growth (Figs. 2–4). The observed differences in the cell proliferation rates of H2122, H157, and Beas2B might be due to the differences in plating efficiencies. Most importantly, we provide mechanistic evidence that KSRP promotes the down-regulation of Spry4 by a previously unidentified mechanism, *i.e.* post-transcriptional mRNA regulation (Figs. 4–6). Taken together, these studies form the preclinical framework to determine whether KSRP represents a suitable future molecular therapeutic target for treating lung cancer.

Although we have demonstrated that KSRP was up-regulated in lung cancer (Fig. 1), several questions still remain to be resolved. It was shown earlier that Dishevelled 3 (Dvl3), a critical regulator of Wnt signaling, regulates β-catenin transcript levels via the interaction with KSRP (11). In another study, Wnt7a/Fzd9 signaling was shown to regulate the expression of Spry4 in NSCLC (9). Based on these observations, it is tempting to speculate that Wnt7a/Fzd9 signaling-mediated regulation of Spry4 might be KSRP-dependent. However, how Wnt7a and/or its signaling intermediates, *i.e.* Dvl3, MEK5, ERK5, and peroxisome proliferator-activated receptor-γ, impair the mRNA decay-promoting abilities of KSRP remains obscure at the level of fine detail and is the subject of future study. Another unanswered research question includes deciphering the mechanism(s) that trigger KSRP expression in response to cigarette smoke.

We have identified a novel role for KSRP in the regulation of cell migration and invasion, which play an indispensable role for cancer metastasis. Spry4 is a demonstrated player that regulates cell migration and invasion in NSCLC (9). Therefore, the influence of KSRP on cell migration and invasion might be through the regulation of Spry4. The observations from the current study suggest that regulated expression of KSRP in the bronchial epithelium leads to the optimal expression of Spry4, which functions as a brake to cell proliferation, cell migration, and invasion. On the contrary, up-regulation of KSRP in NSCLC leads to the destabilization of Spry4 transcripts, resulting in increased cell proliferation, cell migration, and invasion, which contribute to the development of lung cancer.

## Experimental procedures

### Cell culture

Human non-transformed bronchial epithelial cell lines (Beas2B) and NSCLC cell lines (A549, H2122, H157, H661, H358, H23, H1299, H1793, and Calu1) were obtained from the tissue culture core facility of the University of Colorado Anschutz Medical Campus. Beas2B, H157, and H2122 were cultured in RPMI 1640 medium supplemented with 10% FBS in a humidified 5% CO_2_ incubator at 37 °C. HBEC, obtained from Dr. John Minna (University of Texas South Western), was cultured in bronchial epithelial cell growth medium (BEGM^TM^, Lonza). All the cell lines were cultured biweekly, and stocks of cell lines were passaged no more than 10 times for use in experiments.

For generating H2122 clones with stable expression of non-targeting shRNAs and KSRP shRNAs, H2122 cells were transfected with either pENTR/Lacz-control shRNA or pENTR/KSRP shRNA vectors followed by treatment with 100 or 200 μg/ml Zeocin (R25001, Invitrogen). Several Zeocin-resistant clones were subsequently isolated and screened for KSRP knockdown via immunoblotting.

### Immunohistochemistry and tissue microarray

Lung cancer tissue microarrays with 90 cases of lung cancer and 10 normal tissues that were not from the same patient were obtained from Biomax (BC041114, US Biomax Inc., Rockville, MD). The TMAs were deparaffinized, rehydrated with a xylene and graded alcohol series, and subjected to antigen recovery using a citrate-based reagent (H-3300, Vector Laboratories, Burlingame, CA). After blocking nonspecific binding with normal horse serum, the TMAs were subjected to immunostaining with anti-KSRP antibody (HPA034739, Sigma) followed by incubation with ImmPRESS goat anti-rabbit horseradish peroxidase reagent (MP-7401, Vector Laboratories). Visualization was carried out using diaminobenzidine chromogen and then counterstaining with hematoxylin QS (H-3404, Vector Laboratories). Negative controls for immunostaining were performed by omitting the primary antibody and substituting with rabbit IgG. The stained slides were scanned using an Aperio digital pathology slide scanner (Leica Biosystems Inc., Buffalo Grove, IL) and scored using the positive pixel counting algorithm (Leica Biosystems Inc.), and the number of positive pixels were represented in a graph.

### Cell proliferation studies

To measure the cell growth rates, 15,000 cells in complete growth medium were seeded per well in a 24-well culture plate. On subsequent days, cells were trypsinized from the wells with 100 μl of trypsin, diluted with 400 μl of growth medium, and counted using a hemocytometer.

Clonogenic assays were performed in triplicates by seeding 1000 cells/well in a 12-well culture plate followed by incubation at 37 °C in a 5% CO_2_ incubator. After 5–7 days, colonies were stained using a staining solution (0.5% crystal violet, 12% glutaraldehyde, 87.5% H_2_O) for 1 h at room temperature. After destaining in water and drying, colonies were quantified using a Bio-Rad Chemidoc imaging system and Quantity One software.

Cell proliferation was also determined by immunofluorescence staining of incorporated BrdU followed by flow cytometric analysis using a BD Pharmingen BrdU flow kit (FITC BrdU Flow kit, 559619) according to the manufacturer's recommendations. Briefly, H2122 cells were treated with KSRP siRNAs. After 48 h, the cells were pulse-labeled with 1 mm BrdU in PBS for 1 h. After 1 h, the cells were harvested by trypsin digestion followed by staining with FITC-labeled anti-BrdU antibodies and 7-aminoactinomycin D. Analysis was performed using a Gallios^TM^ flow cytometer (Beckman Coulter Life Sciences). The S phase populations were compared and are presented in Fig. 2*J*.

### Cell migration and invasion assays

For assessing cell migration, 15,000 cells in serum-free medium were seeded into Transwell inserts (353097, Corning) containing 8-μm permeable pores and allowed to migrate toward 10% FBS-containing medium. After 16 h, the Transwell inserts containing the cells were removed and washed in PBS three times. The migrated cells on the bottom of the insert were fixed with 5% glutaraldehyde solution followed by crystal violet (1%) staining. After washing the inserts three times with water, the inserts were allowed to air dry, and pictures were taken using an inverted microscope. Ten independent fields were counted for each Transwell, and the average numbers of cells/field were represented in graphs. For assessing cell invasion, 15,000 cells in serum-free medium were seeded into the Matrigel-coated Transwell inserts (BD Biosciences). After 16 h, the invaded cells were stained, counted, and represented in a graph similarly to the migration assay protocol.

### Anchorage-independent growth

Soft agar assays were performed as described previously (8, 41). Briefly, 5000 cells were plated in triplicates in a 6-well plate in a volume of 1.5 ml of growth medium containing 0.3% Noble agar onto a base of 1.5 ml of growth medium containing 0.5% agar (8). The plates were incubated in a 37 °C CO_2_ incubator for 14 days. Later, colonies were stained for 16 h at 37 °C with nitroblue tetrazolium chloride (1 mg/ml), visualized under a microscope, counted, and represented in a graph.

### 3D cell culture

H2122 clones were grown in growth factor-reduced Matrigel (BD Biosciences) basement membrane according to Debnath *et al.* (42). Briefly, 5000 cells/well were grown in 4% Matrigel basement membrane with EGF on a 100% Matrigel layer. After 5–8 days, pictures of the colonies were taken using an inverted microscope equipped with a digital camera. Images were later analyzed by determining the number of spheroids and aggregates, and the spheroid/aggregate ratio was represented in figures.

### RNA immunoprecipitation

Ribonucleoprotein complexes were immunoprecipitated from Beas2B, H2122, and H157 cells according to Bikkavilli and Malbon (11). Briefly, ribonucleoprotein complexes were immunoprecipitated from Beas2B cells expressing either empty vector or FLAG-KSRP using anti-FLAG antibodies, and complexes from H2122 and H157 cells were immunoprecipitated using anti-KSRP antibodies. Later, the RNA from the complexes was isolated using TRIzol reagent (Thermo Fisher). Real-time quantitative PCR amplification was later performed as described under “RNA isolation and real-time PCR.”

### Northwestern assay

Northwestern assays were performed as described earlier (43). Briefly, DIG-labeled 3′-UTR probes of KSRP, GAPDH, and *Spry4* CDS were synthesized *in vitro* using T7 RNA polymerase (Roche Applied Sciences) according to the manufacturer's recommendations in the presence of rNTPs, DIG-UTP, and pcDNA3.1 vectors harboring KSRP or GAPDH UTRs and pCDNA3.1 V5/His TOPO *hSpry4* CDS as templates. For Northwestern analysis, recombinant proteins were resolved by SDS-PAGE and electrophoretically transferred to nitrocellulose membranes (“blots”). The blots were blocked in TBST (50 mm Tris, pH 7.4, 150 mm NaCl, and 0.1% Tween 20) containing 5% not-fat milk for 1 h at room temperature. Later, DIG-labeled probes (1 μg/ml in TBST with milk) were added to the blots and incubated at 4 °C with gentle rocking. After 16 h, the blots were washed thrice in TBST at 5-min intervals. The binding of RNA probes to KSRP was then revealed by probing the blots with anti-DIG alkaline phosphatase fragments diluted (1:1000) in TBST with 5% milk (11093274910, Roche Applied Science) followed by chemiluminescence detection of alkaline phosphatase using CDP-Star (Roche Applied Science).

### Transfections and luciferase reporter assays

The reporter plasmids (pcDNA-Luc and pCDNA-Luc-Spry4 3′-UTR) and CMV-β-galactosidase control plasmids were transiently transfected into cells using Lipofectamine reagent (11668019, Invitrogen) according to the manufacturer's recommendations. After 48 h, the lysates were assayed for luciferase activities. The luciferase values were normalized to β-galactosidase values and represented in graphs.

### KSRP mRNA stability

The half-life of KSRP mRNA was determined as described before (11). Briefly, H157 and H2122 cells treated with either control siRNA or KSRP siRNA were serum-starved for 16 h followed by actinomycin D treatment. Cells were then harvested at the indicated time points after the addition of actinomycin D (5 μg/ml). Total cellular RNA was isolated using TRIzol reagent, and KSRP mRNA levels were quantified by quantitative PCR as described earlier (11). Spry4/actin mRNA remaining (%) *versus* time after actinomycin D treatment was plotted on a semilogarithmic scale, and half-lives were calculated from the slope of fitted lines for each data set. An average of three independent half-life determinations was used for analysis.

### Knockdown protocol

Double-stranded RNAs (siRNAs) targeting human KSRP (5′-GAUCAACCGGAGAGCAAGA-3′) and control siRNAs (5′-UCUGUGAUUUGAAAGACUAGCCAAG-3′) were procured from Invitrogen (22844075) and Qiagen (SI00300587). NSCLC cells were treated with 100 nm siRNAs using Lipofectamine 2000 reagent according to the manufacturer's protocol. Briefly, siRNAs were incubated with 5 μl of Lipofectamine 2000 for 20 min in 200 μl of Opti-MEM medium (Invitrogen), and then the mixture was added into 1 ml of growth medium in a 6-well plate in which cells were cultured to 80% confluence.

### Immunoblotting analysis

Cell extracts were prepared in a lysis buffer (0.5% Triton X-100, 50 mm β-glycerophosphate, pH 7.20, 0.1 mm sodium vanadate, 2 mm MgCl_2_, 1 mm EDTA, 1 mm dithiothreitol, 2 μg/ml leupeptin, and 4 μg/ml aprotinin). The following antibodies were used for immunoblotting: anti-KSRP (HPA034739, Sigma-Aldrich), GAPDH (5174S, Cell Signaling Technology), actin (A3853, Sigma-Aldrich), anti-FLAG (F7425, Sigma-Aldrich), and anti-human Spry4 (AF5070, R&D Systems). Aliquots of various extracts were resolved by 10% SDS-PAGE and transferred to nitrocellulose membranes. The filters were blocked in Tris-buffered saline (10 mm Tris-Cl, pH 7.4, and 140 mm NaCl) containing 0.1% Tween 20 (TTBS) with 3% nonfat dry milk and then incubated with the same blocking solution containing the indicated antibodies at 0.5 μg/ml for 12–16 h. The filters were extensively washed in TTBS, and bound antibodies were visualized with horseradish peroxidase (HRP)-coupled secondary antibodies.

### RNA isolation and real-time PCR

Total RNA from the cells was obtained using TRIzol reagent (10296-010, Invitrogen) according to the manufacturer's recommendations. For quantitative RT-PCR, 3 μg of total RNA was reverse transcribed using random primers, and real-time PCR was performed using the QuantiTect SYBR Green PCR kit (204050, Qiagen) and the Bio-Rad CFX real-time PCR detection system. Each cDNA sample was analyzed in triplicate, and the absolute amounts of transcripts were determined using an external standard. Briefly, a standard curve was generated using Ct values obtained from the real-time PCR using pGFP2-N2-mDvl2 plasmid. The Ct values of the target mRNA were later substituted in the equation from the standard curve to calculate the amount of the amplicon, and the calculated amounts of the amplicons (pg) were represented in graphs. The primers utilized in the PCR experiments were as follows: Spry4-F, 5′-CCAGGATGTCACCCACCATTG-3′; Spry4-R, 5′-TGTGCTGCTGCTGCTC-3′; actin-F, 5′-CCAGCTCACCATGGATGATG-3′; actin-R, 5′-ATGCCGGAGCCGTTGTC-3′; GAPDH-F,5′-CAAGGCTGTGGGCAAGGT-3′; GAPDH-R, 5′-GGAAGGCCATGCCAGTGA-3′; Dvl2-F, 5′-ACGACGATGCTGTACGAGTG-3′; Dvl2-R, 5′-ATTTCGAGGGAGGGTGAAGT-3′. Primers for CD82 were obtained from Integrated DNA Technologies, Inc. (345221533), and the primers for TIMP1 were obtained from SA Biosciences (PPH00771B-200).

### The Cancer Genome Atlas analysis

For determining the expression of KSRP in lung cancer, lung cancer data sets were downloaded from the TCGA data portal (44, 45) and analyzed using the “R” program for statistical computing. For analysis, 103 matched normal-tumor lung cancer data sets were utilized, and the KSRP gene expression values (RNA sequence values) were represented in a graph.

### Exposure of bronchial epithelial cells to CSC

Cultures of human bronchial epithelial cells, *i.e.* Beas2B, were grown to 80% confluence. Treatment with CSC (R100402, Murty Pharmaceuticals, Inc.) at a final concentration of 25 μg/ml was carried out in the culture medium at 37 °C for 4 h. The cell lysates were later probed for the expression of KSRP.

### Exposure of mice to cigarette smoke

C57/BL6 mice were exposed to CS as described earlier (25). Briefly, 8-week-old control group mice were housed in a filtered air environment, whereas the experimental (CS exposure) group was subjected to CS exposure for 5 h/day, 5 days/week for 6 months using a whole-body smoke exposure system (TE-10, Teague Enterprises, Woodland, CA) using 2R4F cigarettes with 2.45 mg of nicotine/cigarette (Tobacco Research Institute, University of Kentucky, Lexington, KY).

### Human tissues

We obtained deidentified human lungs not suitable for transplantation and donated for medical research from the National Disease Research Interchange (Philadelphia, PA) and the International Institute for the Advancement of Medicine (Edison, NJ). Smokers were individuals who smoked 10–25 cigarettes per day for at least 3 years, and non-smokers were individuals who had never smoked. The Committee for the Protection of Human Subjects at National Jewish Health and University of Illinois at Chicago approved this research.

### Data analysis

Data were compiled from at least three independent, replicate experiments, each performed on separate cultures and on separate occasions. Comparisons of data among experimental groups were performed using Student's *t* test or one-way ANOVA as indicated in the figure legends. A *p* value <0.01 is denoted with a ** symbol, and a *p* value <0.05 is denoted with a * symbol.

## Author contributions

RK. B., S. A. Z., M. V. S., L. W., P. Y. W., A. B., S. R., B. A. S., S. A., and R. A. W. designed, performed, and analyzed the experiments. C. D. C. analyzed the TMA slides shown in Fig. 1. N. M. R. and S. P. R. designed and analyzed cigarette smoke exposure in mice shown in supplemental Fig. 1B. B. K. provided the human tissue samples from non-smokers and smokers and analyzed the expression of KSRP shown in supplemental Fig. 1A. K. T. performed the analysis on TCGA data sets shown in Fig. 1*A*. RK. B., S. A. Z., S. A., and R. A. W. wrote the manuscript. All the authors reviewed the results and approved the final version of the manuscript.

## Supplementary Material

Supplemental Data

## Footnotes

2 The abbreviations used are:

ARE-BP: AU-rich element-binding protein
KSRP: K-homology splicing regulatory protein
ARE: AU-rich element
TCGA: The Cancer Genome Atlas
TMA: tissue microarray
NSCLC: non-small-cell lung cancer
HBEC: human bronchial epithelial cell line
CS: cigarette smoke
CSC: cigarette smoke condensate
qPCR: quantitative PCR
DIG: digoxigenin
CDS: coding sequence
Luc: luciferase
Dvl: Dishevelled
ANOVA: analysis of variance.
