# Supplementary material for: K-homology splicing regulatory protein (KSRP) promotes post-transcriptional destabilization of Spry4 transcripts in non-small cell lung cancer
Source: J Biol Chem. 2017 Mar 8;292(18):7423–34. doi: 10.1074/jbc.M116.757906 (PMC5418043; doi:10.1074/jbc.M116.757906)
Supplement: Supplemental Data [file supp_292_18_7423__index.html]

KSRP promotes post-transcriptional destabilization of Spry4 transcripts in non-small cell lung cancer — K-homology splicing regulatory protein (KSRP) promotes post-transcriptional destabilization of Spry4 transcripts in non-small cell lung cancer — KSRP is a novel regulator of Spry4 — Supplemental Data 

# K-homology splicing regulatory protein (KSRP) promotes post-transcriptional destabilization of Spry4 transcripts in non-small cell lung cancer

## Supplemental Data

- Supplementary data (.pdf, 379 KB)
