# Supplementary material for: K-homology splicing regulatory protein (KSRP) promotes post-transcriptional destabilization of Spry4 transcripts in non-small cell lung cancer
Source: J Biol Chem. 2017 Mar 8;292(18):7423–34. doi: 10.1074/jbc.M116.757906 (PMC5418043; doi:10.1074/jbc.M116.757906)

Supplementary Fig. 1. Cigarette smoke induces KSRP expression. ***A.*** KSRP expression in human lung tissue specimens from non-smokers (n=4) and smokers (n=5) were determined by immunoblotting using anti-KSRP antibodies. *, $p < 0.05$, *t*-test. ***B.*** KSRP expression in the lungs of mice exposed to filtered room air (RA, n=3) or cigarette smoke (CS, n=5) for 6 months were determined by immunoblotting using anti-KSRP antibodies. *, $p < 0.05$, *t*-test. ***C.*** KSRP expression in the cell lysates of human non-transformed bronchial epithelial cells (Beas2B) exposed to cigarette smoke condensate (CSC) were determined by immunoblotting using anti-KSRP antibodies. *, $p < 0.05$, *t*-test.

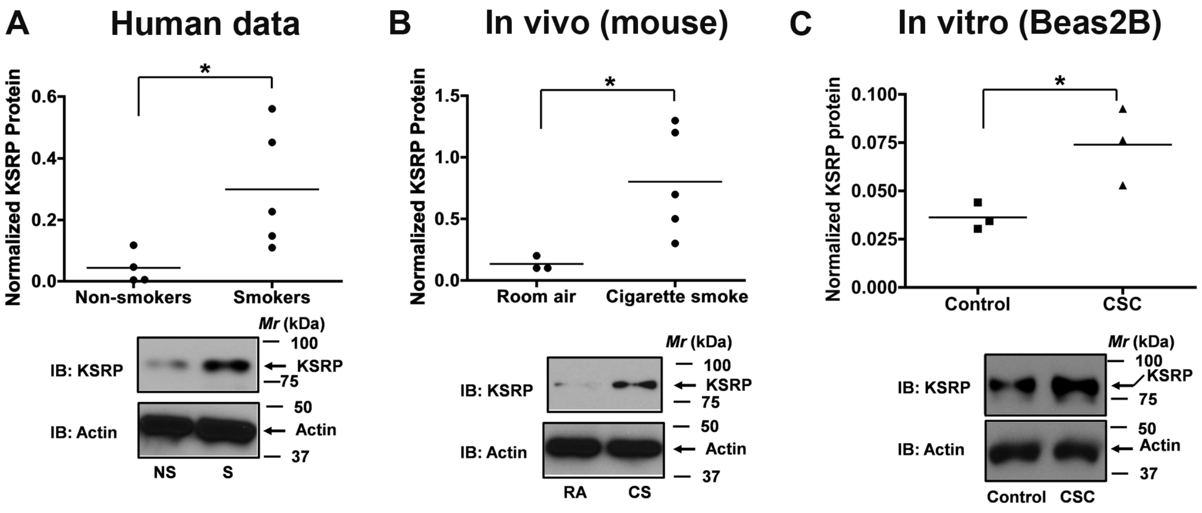

Supplement: Supplemental Data [file 10.1074_M116.757906_jbc.M116.757906-1.pdf]
